# Carbon Dots@rGO Paper as Freestanding and Flexible Potassium‐Ion Batteries Anode

**DOI:** 10.1002/advs.202000470

**Published:** 2020-06-17

**Authors:** Erjin Zhang, Xinxin Jia, Bin Wang, Jue Wang, Xinzhi Yu, Bingan Lu

**Affiliations:** ^1^ School of Physics and Electronics State Key Laboratory of Advanced Design and Manufacturing for Vehicle Body Hunan Provincial Key Laboratory of Multi‐Electron Based Energy Storage Devices Hunan University Changsha 410082 P. R. China; ^2^ Physics and Electronic Engineering Department Xinxiang University Xinxiang 453003 P. R. China; ^3^ College of Chemistry and Chemical Engineering Central South University Changsha 410083 P. R. China; ^4^ Fujian Strait Research Institute of Industrial Graphene Technologies Quanzhou 362000 P. R. China

**Keywords:** carbon dots, flexible anodes, freestanding hybrid architecture, microwaves, potassium‐ion battery anodes, reduced graphene oxide

## Abstract

Carbonaceous materials, especially with graphite‐layers structure, as anode for potassium‐ion batteries (PIBs), are the footstone for industrialization of PIBs. However, carbonaceous materials with graphite‐layers structure usually suffer from poor cycle life and inferior stability, not to mention freestanding and flexible PIBs. Here, a freestanding and flexible 3D hybrid architecture by introducing carbon dots on the reduced graphene oxide surface (CDs@rGO) is synthesized as high performance PIBs anode. The CDs@rGO paper has efficient electron and ion transfer channels due to its unique structure, thus enhancing reaction kinetics. In addition, the CDs provide abundant defects and oxygen‐containing functional groups, which can improve the electrochemical performance. This freestanding and flexible anode exhibits the high capacity of 310 mAh g^−1^ at 100 mA g^−1^, ultra‐long cycle life (840 cycles with a capacity of 244 mAh g^−1^ at 200 mA g^−1^), and excellent rate performance (undergo six consecutive currents changing from 100 to 500 mA g^−1^, high capacity 185 mAh g^−1^ at 500 mA g^−1^), outperforming many existing carbonaceous PIB anodes. The results may provide a starting point for high‐performance freestanding and flexible PIBs and promote the rapid development of next‐generation flexible batteries.

## Graphical abstract

A freestanding and flexible 3D hybrid architecture by introducing carbon dots on the reduced graphene oxide surface is synthesized as a high‐performance potassium‐ion battery anode. It may provide a starting point for high‐performance freestanding and flexible potassium‐ion battery and promote the rapid development of next‐generation flexible batteries.

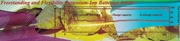

Potassium‐ion batteries (PIBs) are an essential research field for future alternative energy storage devices because of the abundant reserves and high energy density of potassium.^[^ ^1^, ^2^, ^3^, ^4^, ^5^ ^]^ MoSe_2_,^[^ ^6^, ^7^ ^]^ MoS_2_,^[^ ^8^ ^]^ FeS_2_,^[^ ^9^ ^]^ CuS,^[^ ^10^ ^]^ CoS,^[^ ^11^ ^]^ VSe,^[^ ^12^ ^]^ Sb,^[^ ^13^, ^14^ ^]^ and Bi^[^ ^15^ ^]^ have been used for PIBs anode material with a high capacity. However, the performance degradation caused by poor transport kinetic, irreversible alloying effects, and huge volume expansion is unavoidable.^[^ ^16^ ^]^ In contrast, graphite is one of the effective electrode materials with a higher degree of industrialization, more mature processing technology, and lower cost. Graphite can form a stage 1 intercalation compound (GIC, KC_8_) via K ions insertion, resulting in a theoretical capacity of 279 mAh g^−1^.^[^ ^17^, ^18^, ^19^ ^]^ Inferior stability, poor cycle life, and unsatisfactory rate performance are critical obstacles that hinder the practical large‐scale application of graphite anodes. Some strategies such as doping, nanostructures design, and electrolyte engineering have been employed to enhance the electrochemical properties of graphite anodes.^[^ ^20^, ^21^ ^]^ For example, nitrogen‐doped graphene can increase the capacity of PIBs from the theoretical capacity of graphite to over 350 mAh g^−1^.^[^ ^22^, ^23^ ^]^ The hard–soft composite carbon anode achieved capacity retention of 89% after 440 cycles.^[^ ^24^ ^]^ Carbon nanocage and hollow polypyrrole carbon can also achieve the rapid storage of potassium ions.^[^ ^19^, ^25^ ^]^ We have demonstrated graphite as anode for PIBs can be cycling for more than one year (about 1700 cycles) by using 4 m potassium bis(fluorosulfonyl)imide (KFSI) in ethyl methyl carbonate.^[^ ^17^ ^]^ However, when the voltage is higher than 4 V, the KFSI‐based electrolyte will etch the aluminum foil (current collector) and decompose it. In contrast, the traditional low concentration carbonate electrolyte based on potassium hexafluorophosphate (KPF_6_) usually shows high decomposition voltage, high chemical durability, and low cost.

It is known that the carbon dots (CDs) can create an excellent interface for intercalations between electrodes and electrolytes due to the massive flexibility in terms of surface engineering.^[^ ^26^, ^27^, ^28^, ^29^ ^]^ The CDs with the atomic size on a non‐carbon substrate can provide more active sites for ion insertion and extraction, enhancing kinetics, and increasing capacity.^[^ ^30^, ^31^, ^32^ ^]^ Fan et al. have proved that edge‐nitrogen‐rich CDs pillared graphene blocks exhibit ultrahigh capacity and ultra‐long life for sodium storage.^[^ ^33^ ^]^ Park et al. achieved homogeneous Li deposition through the controlling of CD‐assisted Li‐dendrite morphology.^[^ ^34^ ^]^ In fact, PIBs have similar energy storage mechanisms to lithium‐ion batteries and sodium‐ion batteries. Inspired by the above research, we set out to utilize graphene to anchor CDs to cut off the electrical neutrality of carbon materials and fabricate active sites for ions adsorption and desorption, thereby improving the potassium storage performance of the graphite.

Herein, we design a freestanding and flexible carbon‐based composite to improve the electrochemical performance of PIBs by anchoring CDs on the reduced graphene oxide surface (CDs@rGO). The obtained CDs@rGO paper has a 3D structure that enables electrolyte effortlessly to access the active sites and pathways for enhanced electron/ion transfer and diffusion. Density functional theory (DFT) calculations show that the oxygen‐containing functional groups from CDs are favorable for attracting metal cations, thus producing uniform solid electrolyte interphase (SEI). Based on this unique structure, the CDs@rGO anode exhibits a high capacity of 310 mAh g^−1^ at the current density of 100 mA g^−1^ with an initial Coulombic efficiency (ICE) about 44.4%. Operating at a current density of 200 mA g^−1^, it shows an ultra‐long cycle life and retains capacity of 244 mAh g^−1^ after 840 cycles. Besides, an excellent rate performance is achieved (even undergo 6 consecutive currents changing from 100 to 500 mA g^−1^, a capacity of 185 mAh g^−1^ can be delivered at 500 mA g^−1^). Therefore, the CDs@rGO paper may be a promising anode material for PIBs. Moreover, the strategy to enhance the performance of energy storage by introducing carbon quantum dots could apply in other energy fields.

**Figure** **1**a shows the digital photograph of prepared flexible, freestanding CDs@rGO film paper (see Experimental Section, Figure S1, Supporting Information). These thin‐film papers can be tailored to any shape to meet different needs. The top‐view scanning electron microscope (SEM) image of CDs@rGO shows a continuous undulating surface (Figure 1b,c). Fissures occur as the gas remaining in the film breaks through the weak boundary of graphene sheets during the thermal reduction process (Figure S2, Supporting Information). Figure 1d,e exhibits the cross‐section SEM image of CDs@rGO paper. Under the synergy of vacuum filtration and the van der Waals force, graphene sheets are stacked on each other and joined together to form stacked and ordered multilayer structure along the sheet plane. A large number of cavities with different size can store electrolytes (Figure S3, Supporting Information). These special 3D interconnected channels are conducive to electrolyte penetration and ion diffusion in CDs@rGO paper.^[^ ^35^ ^]^ In addition, the graphene sheets are connected vertically and horizontally, so the CDs@rGO paper has a high electrical conductivity, which is conducive to the rapid conduction of electrons in the horizontal and vertical directions.^[^ ^36^ ^]^ Transmission electron microscopy (TEM) image shows a large number of dots located on the surface of the rGO surface (Figure 1f,g). It is because glucose dehydrated to form a C=C bond, forming the basic building block of CDs. Hydrogen and oxygen elements in the hydroxyl group and carboxyl group be dehydrated and removed, while the remaining functional groups will bind to the surface of the CDs and interact with the surface functional groups of GO, thus anchored on GO surface.^[^ ^37^, ^38^ ^]^ Above a certain concentration, CDs are formed. The surface of the pure rGO is clean and free of any load (Figure S4, Supporting Information). This indicates that glucose is converted into amorphous CDs which uniformly grow on the surface of the graphene oxide during the microwave process (Figure S5, Supporting Information).^[^ ^39^ ^]^ Size distribution histogram shows that the average diameter of CDs anchored on graphene oxide is about 2 ± 0.4 nm (Figure 1h). In addition to modifying the surface of rGO, the CD can also act as a binder to ensure the stability of the electrode structure and reduce the influence of the binder on the battery performance (Figure 1i). Moreover, the splicing model as observed in SEM images can also facilitate the conduction of electrons in the vertical and horizontal directions and help relieve mechanical stress. Figure S6 (Supporting Information) shows the X‐ray diffraction (XRD) pattern of GO and rGO. The (0 0 2) peak around 9.1° and (1 0 0) peak around 42.6° can be observed in pristine GO. After thermal reduction, the (0 0 2) characteristic diffraction peak shifted to 21.2° due to the reduced layer spacing.^[^ ^40^ ^]^ From the XRD of the CDs@rGO film paper shown in Figure 1j, two diffraction peaks appear at 21.2° and 26.0°, indicating that there are no other substances except carbon (Figure S7, Supporting Information). For the Raman spectra of CDs@rGO (Figure 1k), the intensity of D band in 1351 cm^−1^ coming from the sp3 carbons defects is significantly higher than the G band in 1594 cm^−1^.^[^ ^41^, ^42^ ^]^ The *I*_D_/*I*_G_ Raman peak ratio for CDs@rGO film paper is 1.61, which is much higher than that of rGO (1.26, Figure S8, Supporting Information). The higher *I*_D_/*I*_G_ value indicates that the introduced CDs can increase the edge of the rGO and thus leads to more active sites, which means a higher capacity can be achieved.^[^ ^43^ ^]^ The oxygen peak in X‐ray photoelectron spectroscopy (XPS) spectra (Figure 1l and Figure S9, Supporting Information) indicates that the introduction of CDs brings more oxygen‐containing functional groups, which is conducive to guiding potassium ions to be uniformly embedded in the graphite layer through electrostatic action.^[^ ^34^ ^]^

**Figure 1:**
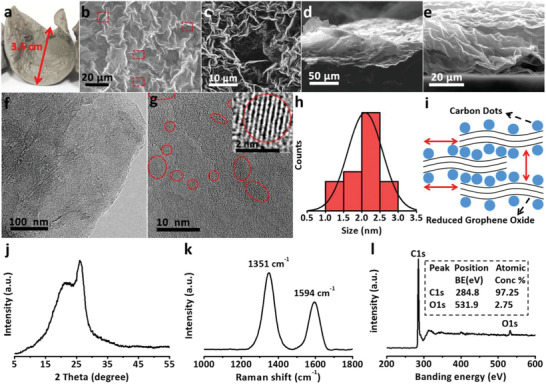
a) Prepared flexible, freestanding CDs@rGO film paper. b,c) Top view SEM images of CDs@rGO film paper (red mark: fissures). d,e) Cross‐section SEM images of CDs@rGO film paper. f) TEM and g) HRTEM images of the CDs@rGO paper. h) The size distribution histogram of CDs anchored on rGO, over 50 CDs were counted for statistics. i) Schematic diagram of CDs@rGO stacked models. j) XRD patterns of CDs@rGO film paper. k) Raman spectra of CDs@rGO film paper. l) The XPS spectra of pristine CDs@rGO film.

The CDs@rGO film paper is assembled with a K foil in a coin cell for the electrochemical property study and the battery performance evaluation. **Figure** **2**a shows the cyclic voltammetry (CV) curves of the CDs@rGO between 0.01 and 3 V at the scan rate of 1 mV s^−1^ for the initial 5 cycles. The charge–discharge profiles in Figure 2b demonstrate a high initial discharge/charge capacity at 100 mA g^−1^ about 698 and 310 mAh g^−1^, respectively. The ICE is about 44%, which is comparable to the reported modified graphite‐based anode and much higher than 15.8% of the rGO paper anode (Figure S10, Supporting Information).^[^ ^23^ ^]^ The initial capacity loss is mainly due to the formation of solid electrolyte interphase (SEI) films caused by the decomposition of the electrolyte.^[^ ^44^ ^]^ The stable SEI is important for achieving high Coulombic efficiency and long cycling stability of K/graphite half‐cells.^[^ ^45^ ^]^ From the second cycle, almost the same curve was observed and a high reversible capacity of 297 mAh g^−1^ was achieved after 100 cycles, incarnating a highly reversible and stable cycling performance. Meanwhile, the d*Q*/d*V* differential profile for the initial 2 cycles further proves the transformation processes (Figure 2c). It is obvious that three main peaks located at 0.81, 0.44, and 0.17 V were observed during the first potassiation process in d*Q*/d*V* differential profile. The peaks at 0.81 and 0.44 V are from the irreversible reaction between potassium and surface functional groups, forming a solid electrolyte interface (SEI) layer with the electrolyte.^[^ ^1^, ^46^ ^]^ The peak at 0.17 V corresponds to the formation of KC_8_.^[^ ^18^ ^]^ During de‐potassiation process, an obvious peak at 0.25 V and a feeblish peak at 0.48 V could be distinguished, indicating the gradual disappearance of the graphite intercalation compound. During the second cycle, the cathodic peak at 0.17 V and anodic peak at 0.25 V correspond to the K^+^ intercalation/de‐intercalation reaction.

**Figure 2:**
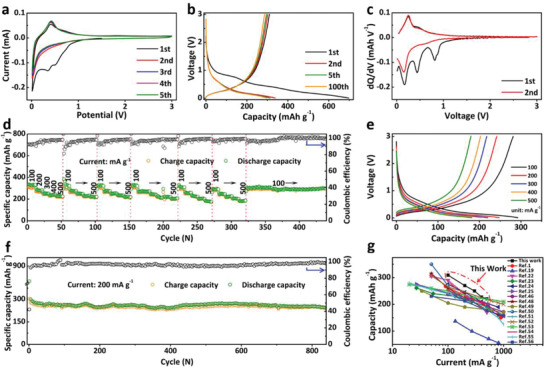
a) CV curves at the scan rate of 1 mV s^−1^. b) Charge–discharge profile at 100 mA g^−1^. c) Corresponding d*Q*/d*V* profiles of the initial 2 cycles. d) Rate performance at various current densities from 100 to 500 mA g^−1^ after activation at 100 mA g^−1^. e) Corresponding charge–discharge profiles at 100–500 mA g^−1^. f) Long‐term cycling performance at 200 mAh g^−1^. g) Comparison of the specific capacity under different currents between the CDs@rGO film paper and other reported carbonaceous electrodes for PIBs.

For manufacturing, the high rate performance is a critical feature to achieve the high‐power‐type KIBs. When the current densities increase from 100 to 200, 300, 400, and 500 mA g^−1^, the CDs@rGO film has a reversible capacity of 309, 270, 250, 227, and 221 mAh g^−1^, respectively (Figure 2d). Even after 6 consecutive current changes, a capacity of 185 mAh g^−1^ (about 60% retention of the capacity at 100 mA g^−1^) can be delivered at 500 mA g^−1^. When the current density returns to 100 mA g^−1^, the battery can still provide a discharge capacity of 303 mAh g^−1^ and safely operated more than 100 cycles with a Coulombic efficiency of 99%. In the same case, the rGO electrode only delivered a discharge capacity of 199, 86, 39, 15, and 5 mAh g^−1^, respectively (Figure S11, Supporting Information). Even if the current is reduced to 100 mA g^−1^, this electrode can only provide a discharge capacity of 220 mAh g^−1^ with a CE of 58%. The charge–discharge profiles of CDs@rGO anode under different current densities are shown in Figure 2e. Only a slight polarization was observed, indicating that the electrode has excellent ion transmission ability and superior rate capability. Except for the superior rate behavior, the CDs@rGO film paper delivered an excellent cycling performance with the extremely high capacity and ultra‐long cycle life. Figure S12 (Supporting Information) shows the cycle performance of CDs@rGO anode at 100 mA g^−1^. The CDs@rGO film paper delivered a high capacity of 311 mAh g^−1^ after 280 cycles without reduction decrease. The extremely high capacity may be due to the large number of defects introduced by the carbon dots.^[^ ^22^, ^47^ ^]^ Benefiting from the unique stacking structure of CDs@rGO anode, the battery possesses a high discharge capacity of 244 mAh g^−1^ after 840 cycles at the current density of 200 mA g^−1^ with a high CE about 98% (Figure 2f). From the second cycle, the average reversible capacity decay rate per cycle is only 0.03%. In contrast, the rGO film electrode delivered an initial discharge capacity of 808 mAh g^−1^ at 100 mA g^−1^, after 100 cycles, the discharge capacity sharp decrease to 84 mAh g^−1^ with a CE about 93% (Figure S13, Supporting Information). Even compared with other state‐of‐the‐art carbon‐based potassium battery negative electrodes, the CDs@rGO film is highly competitive in specific capacity, discharge plateau, and cycle performance (Table S1, Supporting Information).^[^ ^1^, ^19^, ^22^, ^23^, ^24^, ^25^, ^46^, ^48^, ^49^, ^50^, ^51^, ^52^, ^53^, ^54^, ^55^, ^56^ ^]^ The rate performance is also better than most heteroatom‐doped or special structure carbonaceous anodes (Figure 2g). Such an outstanding cycle life, high capacity retention, gratifying rate performance, and high Coulombic efficiency make the CDs@rGO film a prospective anode material for KIBs.

To further investigating the origin of the superior electrochemical performance of the CDs@rGO electrode, CV techniques and electrochemical impedance spectroscopy (EIS) were measured. **Figure** **3**a shows similar CV curves with redox peaks of the CDs@rGO electrode at various sweep rates ranging from 0.1 to 0.4 mV s^−1^, which are used to investigate the diffusion process. The relationship between the measured current (*i*) and the sweep rate (*v*) is used to analyze the capacitive effect. In *i = av^b^*, where *a* and *b* are positive variables, the *b*‐value can be determined from the slope of the plot of log (*i*) versus log (*v*). It is reported that *b* = 0.5 represents a Faradaic intercalation process and the current is diffusion controlled, while for the capacitive response where *b* is close to 1. As shown in Figure 3b, the *b*‐value of the cathodic peak is 0.56, slightly higher than 0.5, suggesting that the diffusion process dominates the current. However, when plotting *b*‐values as a function of potential in the cathodic scan (Figure S14, Supporting Information), the *b*‐values are in the range of 0.68–0.95 at potentials higher than 0.4 V, and the *b*‐values are close to 0.5 at potentials lower than 0.4 V. The result suggests that the response current related to the capacitive exists throughout the reaction, and it is more intense in the electrochemical process above 0.4 V. As shown in Figure 3c, at the sweep rate of 0.1 mV s^−1^, 36.9% of the total charge storage capacity of the CDs@rGO electrode is capacitive based on the integration of CV curves. Although the charge storage capacity comes primarily from the K‐ion intercalation, the role of capacitive contribution further enlarges with a maximum value of 55.2% at 0.4 mV s^−1^ as the scan rate increases (Figure 3d). When continuing to speed up the scan rate, the CV curves show a quasi‐rectangular shape, indicating the singular rate capability of CDs@rGO (Figure S15, Supporting Information). The high proportions of pseudo‐capacitance contributions come from fast kinetics, thus the CDs@rGO electrode exhibits a good rate capacity as an anode for KIBs under high current density.

**Figure 3:**
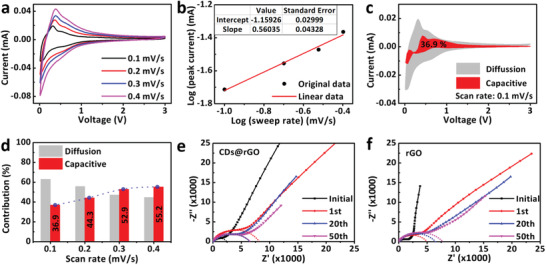
a) The CV curves for CDs@rGO half‐cells from 0.1 to 0.4 mV s^−1^. b) *b*‐value determination for the peak cathodic currents. c) Capacitive (red) and diffusion‐controlled (gray) contribution of the CDs@rGO electrode to charge storage at 0.1 mV s^−1^. d) Normalized contribution ratio of capacitive (red) and diffusion‐controlled (gray) capacities at different scan rates. e) The EIS tests of K/CDs@rGO batteries. f) The EIS tests of K/rGO batteries.

We employed the EIS technique to characterize the charge transfer behavior of potassium ion in K/CDs@rGO half‐cells after a specific cycle number at full charge state. As shown in Figure 3e, each plot contains one semicircle at the high‐medium frequency and one oblique line at the low‐frequency region, which is assigned to charge transfer resistance and the diffusion of cations, respectively. For the initial plot, the fast increase of the imaginary part at low frequency is the typical behavior of the bulk‐type electrode without a charge transfer reaction. After 1 cycle, charge transfer resistance increases sharply, which is related to the formation of the SEI film and capacitive effect.^[^ ^19^ ^]^ Moreover, in Figure S16 (Supporting Information), a smaller impedance originated from the decrease of the charge transfer resistance, indicating that the reaction kinetics accelerates as the reduction reaction processed.^[^ ^23^ ^]^ The charge transfer resistance decreased slightly after 20 cycles and did not increase substantially after 50 cycles, implying a stable SEI film formation on the surface of the CDs@rGO electrode. In contrast, the charge transfer resistance increases from the first cycle to the 50th cycle, indicating that SEI film continues to grow on the rGO electrode/electrolyte interface (Figure 3f). This result is consistent with the low Coulombic efficiency of the K/rGO half‐cells. The stable SEI film and fast reaction kinetic guarantee the excellent cycle stability and outstanding rate performance for the CDs@rGO electrode.

To confirm the K ion storage mechanism and structural stability of CDs@rGO anode, the morphology change and surface composition are investigated. **Figure** **4**a‐c shows the contour plot of the operando XRD results and corresponding waterfall representation XRD patterns for the first two cycles. During the first discharge process, the peak (0 0 2) is gradually weakened, and a new characteristic peak appears at 33.4°. With the progress of the charging process, the characteristic peak at 33.4° gradually disappeared, and the (0 0 2) peak increases significantly. This change indicates that during the discharge, K ions are gradually embedded into the graphite layer and eventually form KC_8_. In the subsequent charging process, K ions are extracted from the graphite. In the second discharge and charge process, the (0 0 2) peak disappears and reappears periodically as in the first cycle. It is worth mentioning that the (0 0 2) peak intensity does not decrease and even did not completely disappear after the first discharge. This is due to the formation of the SEI film based on the KPF_6_ traditional electrolyte during the embedding process. This phenomenon was hardly observed in the third and four cycles, indicating the formation of the stable SEI film (Figure S17, Supporting Information).

**Figure 4:**
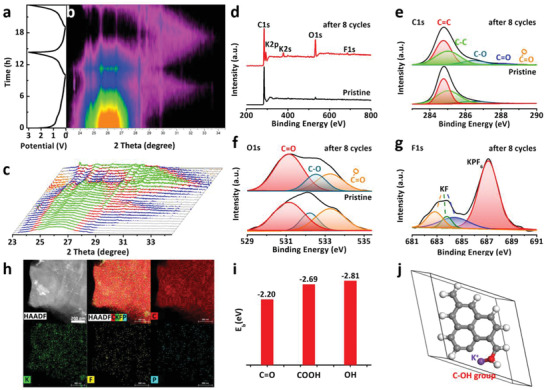
a–c) Contour plot of the operando XRD results of CDs@rGO film anode during discharging/charging process for the initial three cycles. The XPS spectra of pristine CDs@rGO film anode and anodes from half‐cell after 8 cycles: d) Full survey XPS; e) C1s XPS; f) O1s XPS; g) F1s XPS. h) C, K, F, and P elements distribute from full charged CDs@rGO electrode. i) The binding energy of the K ions with O‐related groups. j) Model structures for the binding energy calculation of a K ion and C–OH groups.

Furthermore, we employed X‐ray photoelectron spectroscopy (XPS) analysis to investigate the contents of elements change on CDs@rGO anode surface at pristine and after 8 cycles. As observed in Figure 4d, the survey XPS spectrum indicates that a large number of K and F elements appear on the electrode surface after 8 cycles in addition to the C and O elements. According to the atom concentration list in Table S2 (Supporting Information), the content of element O increases from 2.75% to 17.76% after 8 cycles for the pristine electrode. These results imply the existence of quite differences in the chemical structure of the electrode surface before and after 8 cycles. In general, the peaks of C=C (284.75 eV), C—C (285.1 eV), C—O (286.54 eV), and invisible C=O (289.04 eV) exhibit in high‐resolution C1s of pristine CDs@rGO electrode (Figure 4e). After 8 cycles, a higher proportion of C—O and C=O appears on the electrode surface. Even O—C=O bond appears at 291.19 eV. Similarly, the ratio of C=O bond and O—C=O bond increases obviously compared with the O1s spectrum of the pristine electrode, which may correspond to the decomposition of ethylene carbonate (EC) or dimethyl carbonate (DMC) solvents (Figure 4f).^[^ ^44^ ^]^ The oxygen ratio of 17.76% resulting that the decomposition of the solvent produces an organic SEI film. It is worth noting that in the high‐resolution F1s XPS (Figure 4g), in addition to the P—F bonds, there are a large number of F—K bonds and F—C bonds, which may be derived from the decomposition of KPF_6_ salt and additive agent (fluoroethylene carbonate, FEC). The increase of F element content can inhibit further decomposition of the carbonic acid solvent as previously reported, which is advantageous for the formation of the stabilizing SEI layer.^[^ ^57^, ^58^ ^]^ Figure S18 (Supporting Information) shows the full discharge state TEM images of CDs@rGO electrode after 100 cycles at 100 mA g^−1^. Abundant potassium indicates that K ions are embedded in the electrode during the discharge process. At the full charge state, Figure 4h shows the well‐dispersed C, K, F, and P elements from the stable electrode/electrolyte interface, which is the nature of extremely high electrochemical stability. Importantly, as Table S3 (Supporting Information) shows, the K element content is reduced to 5%, while the F and P elements are identical to the full charge state, indicating that a stable SEI layer was formed on the electrode surface during cycling. The stable SEI highly increases the cycle life. In addition, Figure S19 (Supporting Information) shows the TEM images at different magnifications after cycling. The large‐scale well‐structured reduced graphene oxide sheet proves that the CD@rGO electrode can maintain the structural integrity after potassium ion insertion and extraction, which provides a guarantee for long cycle life. We calculated the binding energy of the interaction between the K ions and O‐related groups of the CDs by the DFT method. As shown in Figure 4i,j, and Figure S20 (Supporting Information), the binding energy of functional groups such as carbonyl, carboxylic, and oxhydryl with K ions are −2.20, −2.69, and −2.81 eV, respectively. The results show that negatively charged electrodes are more attractive to K cations, which has also been verified in lithium and sodium batteries.^[^ ^40^, ^59^ ^]^ Compared to the rGO electrode, CDs@rGO electrodes contain more oxygen‐containing functional groups, which is more conducive to attract K ions through strong interactions and avoid the generation of dendrites.

Flexible batteries have attracted attention as a necessary power source in flexible and wearable electronic devices. Figure S21a (Supporting Information) shows the digital photograph of K/CDs@rGO pouch cell in the folding state under open‐circuit voltage. When the battery was flattened, the voltage showed little change (Figure S21b, Supporting Information). When the battery was folded in half for 20 times in a row, the open‐circuit voltage remained unchanged (Figure S21c, Supporting Information). The freestanding CDs@rGO electrode remained intact, indicating their excellent mechanical strength flexibility (Figure S21d, Supporting Information). The flexible electrode will be widely used and influence future battery systems.

To summarize, we have designed a freestanding and flexible pure carbon‐based composite through anchoring CDs on the surface of reduced graphene oxide. The tailorable CDs@rGO paper with 3D‐structure can improve the kinetics by effectively shortening the electron and ion conduction distances. The introduction of CDs with oxygen‐containing functional groups is conducive to the orderly induction of K ions to form a stable SEI on the electrode surface and maintain the integrity of the electrode structure. Consequently, the battery exhibits a measured specific capacity of 310 mAh g^−1^ at 100 mA g^−1^ with ICE about 44%. After 840 times at current of 200 mA g^−1^, a capacity of 244 mAh g^−1^ with 0.03% average decay rate per cycle is achieved. Moreover, CDs@rGO paper shows a superior rate performance (undergo 6 consecutive current changes from 100 to 500 mA g^−1^, retain the capacity of 185 mAh g^−1^ at 500 mA g^−1^) than the anodes of other carbon‐based PIBs. Cyclic voltammetry, operando XRD measurements, XPS analysis, and DFT calculation were used to systematically study the reaction mechanism. Our strategy that the introduction of quantum dots might be extended to other battery systems to develop stable and long cycle‐life rechargeable batteries.

## Experimental Section

##### Preparation of CDs@rGO Paper

First, 20 mg GO was dissolved into 20 mL distilled water by an ultrasonic concussion to form a clear solution. Then, 1 g glucose was added to the solution and stirred for 10 h until completely dissolved. Then the solution was put into a domestic microwave oven (700 W) for 5 min. The product was ultrasonically dispersed in 100 mL of deionized water and filtrated by vacuum using an ordinary filtration membrane. The freestanding binder‐free flexible paper was peeled off from the filtration membrane after being dried 2 h in a vacuum. Finally, the composite paper was annealed at 1000 °C for 2 h with a heating rate of 10 °C min^−1^ under H_2_/Ar (5%) atmosphere. Thus, the freestanding binder‐free flexible CDs@rGO paper was prepared.

rGO paper was prepared by dissolving GO into distilled water and using vacuum filtration directly after ultrasonic. Then it was annealed at 1000 °C for 2 h with a heating rate of 10 °C min^−1^ under H_2_/Ar (5%) atmosphere.

##### Material Characterization

CDs@rGO paper was characterized by field‐emission SEM and TEM. Powder XRD data were obtained using a Bruker D8 ADVANCE (Cu K*α*). A Thermo Fisher Scientific (K‐alpha 1063) was adopted for XPS measurements. Raman spectroscopy was obtained using WITec (alpha 300 R with a 532 nm wavelength yttrium aluminum garnet (YAG) laser).

##### Electrochemical Measurements

Coin cells (CR2032) were assembled in an argon‐filled glove box (<1 ppm of water and oxygen). CDs@rGO paper directly served as anode, and the potassium metal was cut into pieces as the counter electrode. The composition of electrolyte is 0.8 m KPF_6_ in ethylene carbonate (EC)/dimethyl carbonate (DMC) + 5 wt% fluoroethylene carbonate (v/v = 1:1). The assembled coin cell was tested at various current densities over the range of 0.01–3.0 V with Neware BTS‐53 battery testing system. CV measurements were performed on a CHI660e electrochemical workstation (Chenhua, Shanghai) at 0.1 mV s^−1^ from 0.01 to 3.0 V.

##### Calculated Method

The present calculations were performed based on the DFT within the Cambridge Serial Total Energy Package (CASTEP) plane wave code.^[^ ^60^, ^61^, ^62^ ^]^ Norm conserving pseudopotentials were used to describe the interaction of ionic core and valence electrons. Valence states were considered in this study corresponding to C2s^2^p^2^, O2s^2^p^4^, and K4s^1^. The generalized gradient approximation (GGA) of Perdew–Burke–Ernzerh method parameterized by Perdew was used to calculate the exchange and correlation terms.^[^ ^63^ ^]^ Brillouin‐zone integrations were performed using Monkhorst and Pack *k*‐point meshes.^[^ ^64^ ^]^ During the calculation for graphite, the 600 eV for cutoff energies and 5 × 5 × 1 for the numbers of *k*‐point can ensure the convergence for the total energy. All the calculations were considered converged when the maximum force on the atom was below 0.05 eV Å^−1^, maximum stress was below 0.1 GPa, and the maximum displacement between cycles was below 0.002 Å.

##### Statistical Analyses

Statistical analysis was performed using Nano Measurer software. Over 50 CDs were counted for statistics. All data were presented as means ± SD.

## Conflict of Interest

The authors declare no conflict of interest.

## Supporting information

Supporting Information
