# Supplementary material for: Carbon Dots@rGO Paper as Freestanding and Flexible Potassium‐Ion Batteries Anode
Source: Adv Sci (Weinh). 2020 Jun 17;7(15):2000470. doi: 10.1002/advs.202000470 (PMC7404153; doi:10.1002/advs.202000470)
Supplement: Supplementary file 1 — Supporting Information [file ADVS-7-2000470-s001.pdf]

© 2020 Wiley-VCH GmbH

ADVANCED SCIENCE
Open Access

Supporting Information

for *Adv. Sci.*, DOI: 10.1002/advs.202000470

Carbon Dots@rGO Paper as Freestanding and Flexible Potassium-Ion Batteries Anode

*Ejin Zhang, Xinxin Jia, Bin Wang, Jue Wang, Xinzhi Yu, and Bingan Lu**

Supporting Information

Carbon Dots@rGO Paper as Freestanding and Flexible Potassium-Ion Batteries Anode

Ejin Zhang, Xinxin Jia, Bin Wang, Jue Wang, Xinzhi Yu, and Bingan Lu[*]

Dr. E. Zhang, Dr. X. Jia, Dr. X. Yu, Prof. B. Lu
School of Physics and Electronics, State Key Laboratory of Advanced Design and Manufacturing for Vehicle Body, Hunan Provincial Key Laboratory of Multi-electron based Energy Storage Devices, Hunan University, Changsha, 410082, PR China

Dr. B. Wang
Physics and Electronic Engineering Department, Xinxiang University, Xinxiang, 453003, PR China

Dr. J. Wang
College of Chemistry and Chemical Engineering, Central South University, Changsha, 410083, PR China

Prof. B. Lu
Fujian Strait Research Institute of Industrial Graphene Technologies, Quanzhou, 362000, PR China

[*]E-mail: *luba2012@hnu.edu.cn (B. Lu)*

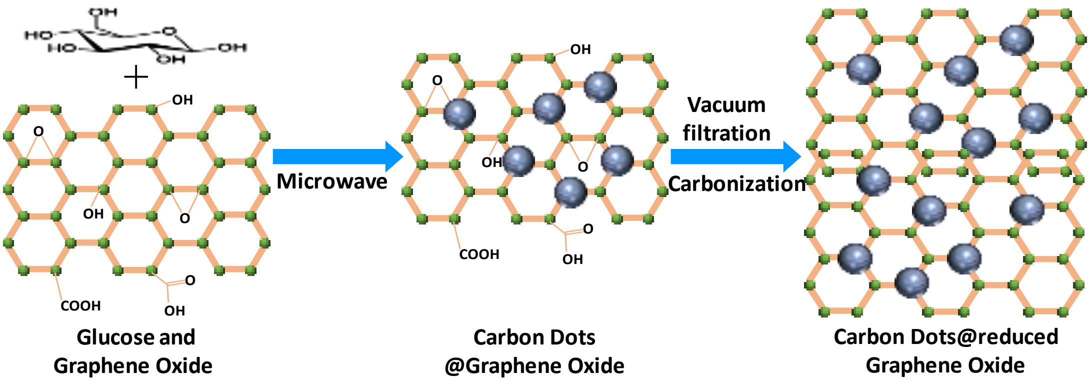

Figure S1. Schematic illustration of the fabrication process for the freestanding Carbon Dots@reduced graphene oxide (CDs@rGO) paper.

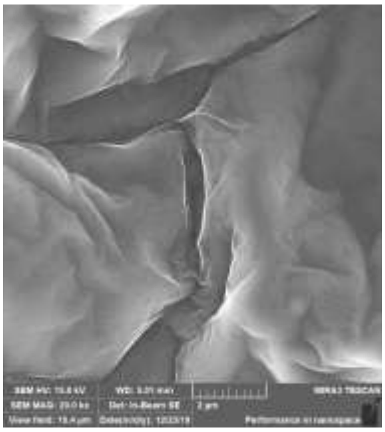

Figure S2. Fissure at the CDs@rGO paper surface.

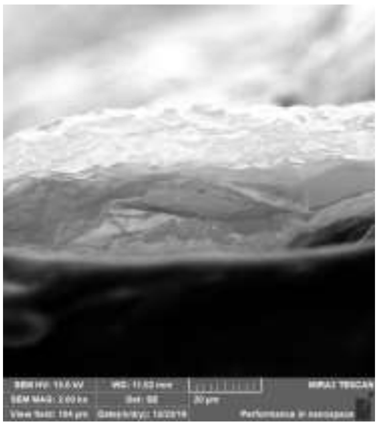

Figure S3. Cavities in the CDs@rGO paper.

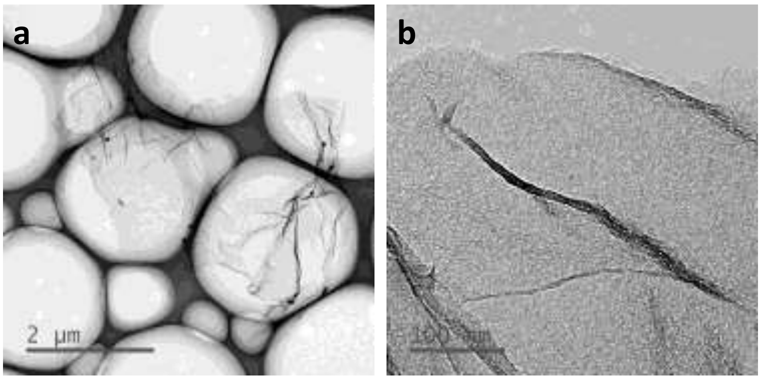

Figure S4. a) and b) TEM images of the rGO.

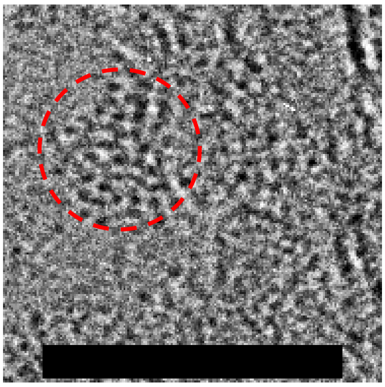

Figure S5. HR-TEM image of the CDs. Scale bar 5 nm.

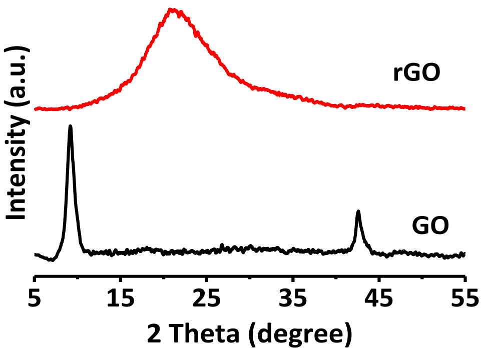

Figure S6. XRD patterns of GO and rGO.

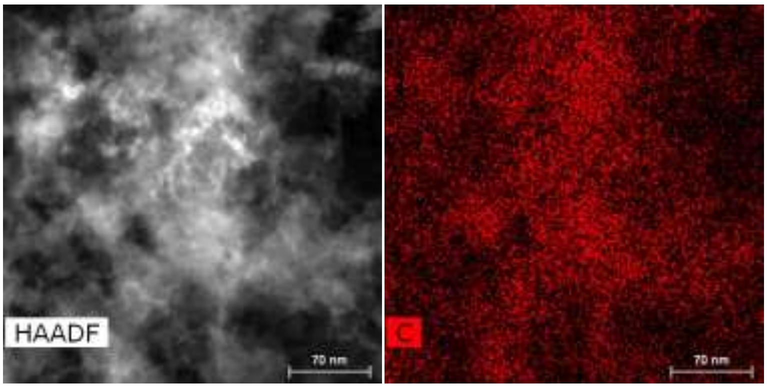

Figure S7. a) and b) EDX mapping image of the CDs@rGO.

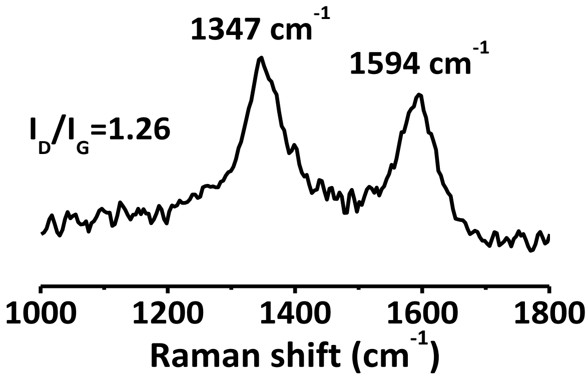

Figure S8. Raman spectra of rGO.

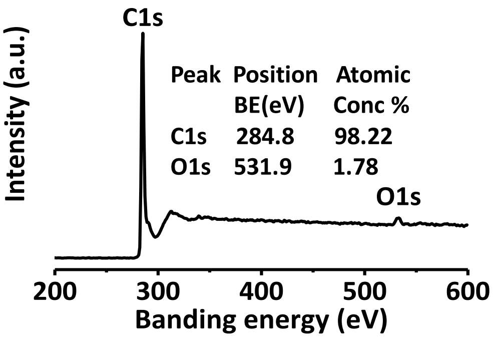

Figure S9. The XPS spectra of pristine rGO.

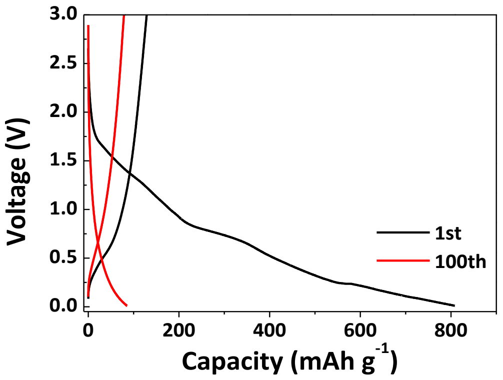

Figure S10. Charge-discharge profiles of rGO anode at 100 mA g^{-1}.

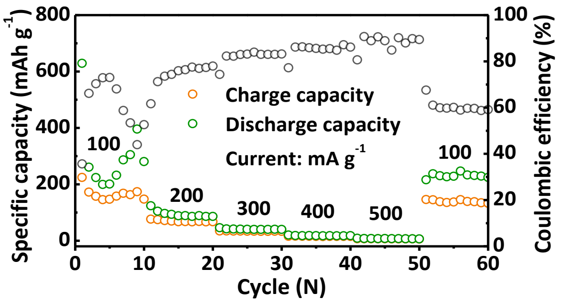

Figure S11. Rate performance for rGO anode at various current densities from 100 to 500 mA g^{-1}.

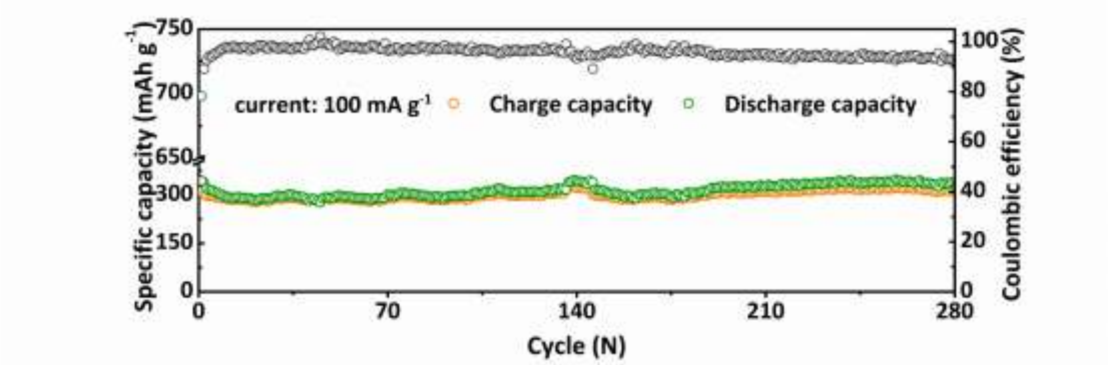

Figure S12. The cycle performance of CDs@rGO anode at 100 mA g^{-1}.

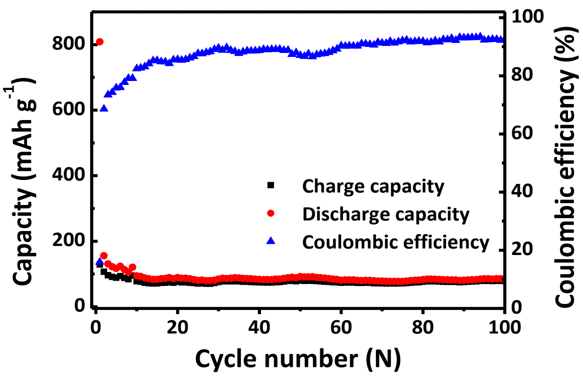

Figure S13. Cyclic performance of rGO anode at 100 mA g^{-1}.

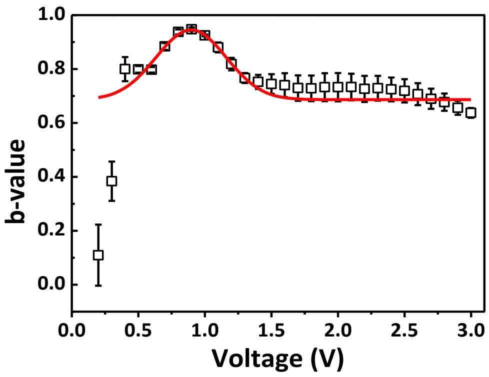

Figure S14. *b*-values as a function of potential for the cathodic scan (K^+ intercalation).

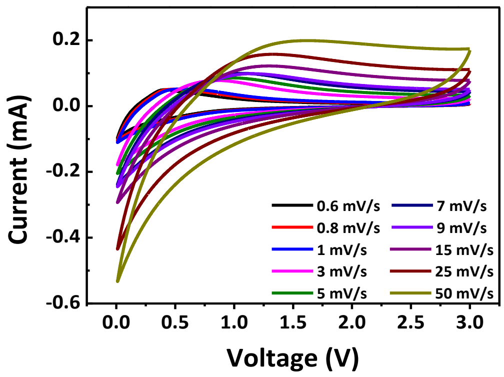

Figure S15. CV curves of K/CDs@rGO half-cell at high scan rates.

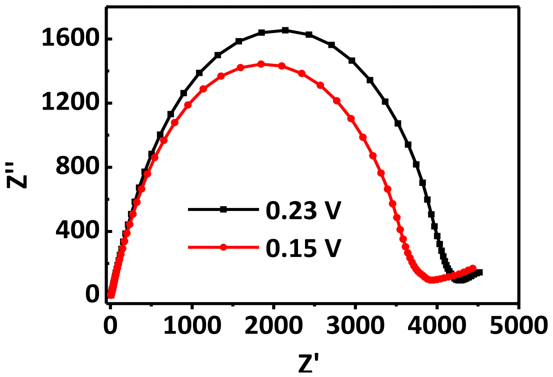

Figure S16. EIS analysis at different discharge potentials.

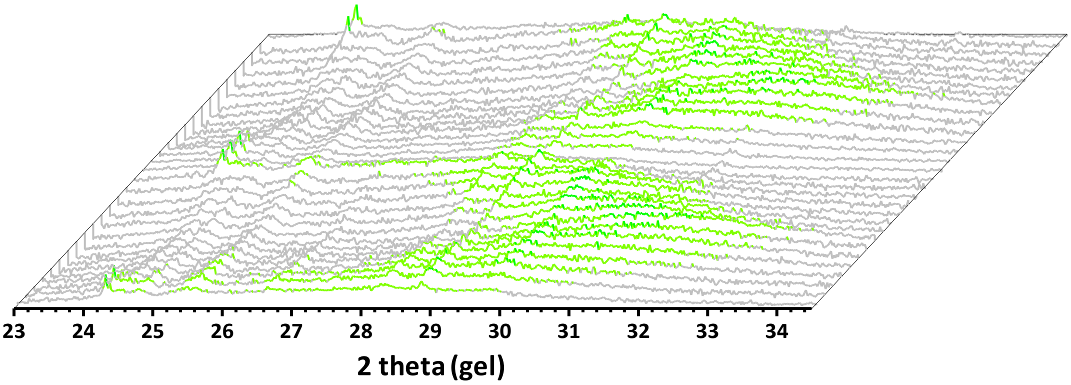

Figure S17. Waterfall representation operando XRD patterns for third and fourth cycle.

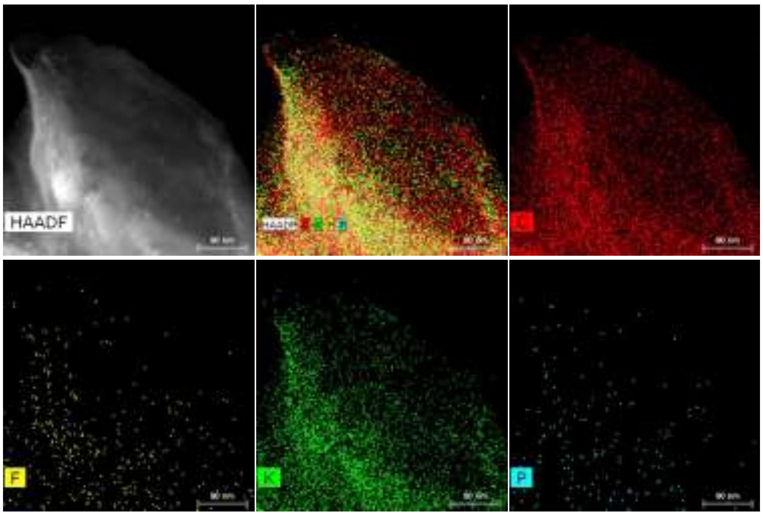

Figure S18. C, K, F, and P elements distribute from full discharged CDs@rGO electrode.

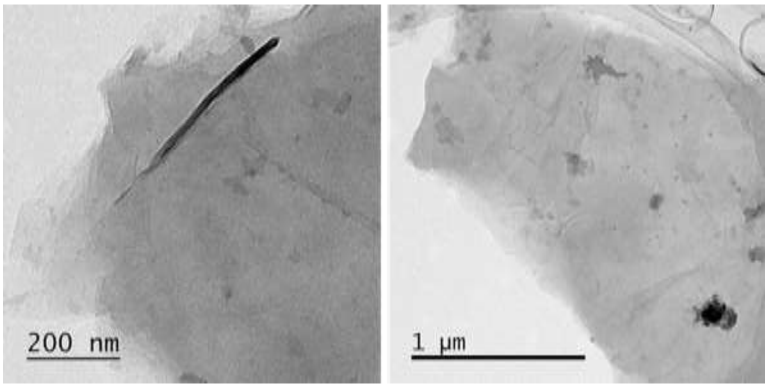

Figure S19. TEM images at different magnifications after cycling.

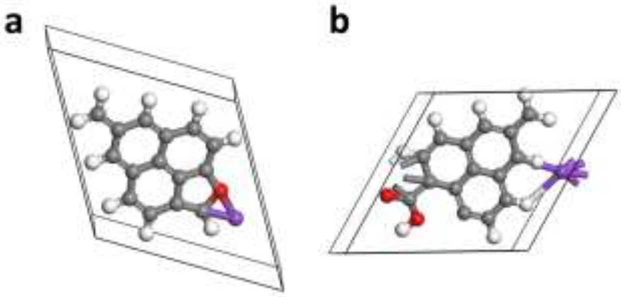

Figure S20. Model structure for the binding energy calculation of a K ion with COOH and C=O.

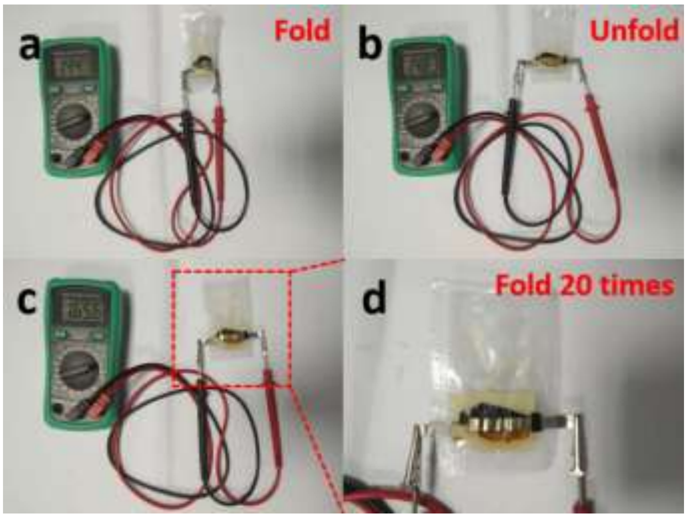

Figure S21. Digital photograph of K/ CDs@rGO pouch cell at a) fold and b) unfold state; c and d) after fold 20 times.

Table S1. The specific capacity, charge plateau, and cycle-life between the CDs@rGO electrode and other reported carbonaceous electrodes for PIBs.

Positive Electrode Materials	Current Density (mA g^{-1})	Cycle number	Initial Capacity (mAh g^{-1})	Capacity Retention	Initial CE	Charge Plateau (V)
3D rGO Aerogel	90	100	294	78%	44%	0.3,0.6
Carbon Nanocage	56	100	212	92%	40%	<0.6
Few-layer Nitrogen-doped Graphene	50<br>500	60<br>500	352.2	90%<br>150	37%	
Hard–Soft Carbon nanotube	289<br>100	200<br>500	200<br>210	93%<br>90%	67%<br>~18%	
Multishelled Hollow Carbon Nanospheres	558	100	212	95%		
N-doped carbon nanofibers	25<br>2000	 4000	248<br>104		49%	0.25
N/O dual-doped hierarchical Porous Hard Carbon	1050	1100	130	69.5%	25%	
Nitrogen-doped Carbon Microsphere	33.6<br>500	200<br>4000	250<br>180	82%		
Nitrogen-doped Few-Layer Graphene	100	100	270	77.8%	~50%	
Nitrogen-doped Porous Carbon	1000	2500	170	88%		
N-doped Carbon	100<br>1000	100<br>800	301.8<br>190	84%<br>84%	37%	
Porous CNF Paper	200	1200	239	88%	24.1%	0-1
Potato Biomass Porous Carbon	500	400	224	87.5%	42%	
Nitrogen-doped Carbon Nanotubes	2000	500	131	77.86%		

Table S2. The atomic concentration of elements of CDs@rGO anode from half-cell obtained from XPS analysis (%).

	C	O	K	F	P
Pristine	97.25	2.75	0	0	0
After 8 cycles	74.77	17.76	5.9	1.35	0.21

Table S3. The atomic concentration of elements of CDs@rGO anode from half-cell obtained from EDS (%) at full charge and full discharge state.

	C	K	F	P
Full charge	93.34	5.14	1.07	0.45
Full discharge	40.74	57.91	0.72	0.63
